# A Putative Role of de-Mono-ADP-Ribosylation of STAT1 by the SARS-CoV-2 Nsp3 Protein in the Cytokine Storm Syndrome of COVID-19

**DOI:** 10.3390/v12060646

**Published:** 2020-06-15

**Authors:** Jean-Michel Claverie

**Affiliations:** Structural & Genomic Information Laboratory (IGS, UMR 7256), Mediterranean Institute of Microbiology (FR3479), Aix-Marseille University and CNRS, 13288 Marseille, France; claverie@igs.cnrs-mrs.fr

**Keywords:** de-MARylation, interferon-stimulated gene, inflammation, PARP14, ACE2

## Abstract

As more cases of COVID-19 are studied and treated worldwide, it had become apparent that the lethal and most severe cases of pneumonia are due to an out-of-control inflammatory response to the SARS-CoV-2 virus. I explored the putative causes of this specific feature through a detailed genomic comparison with the closest SARS-CoV-2 relatives isolated from bats, as well as previous coronavirus strains responsible for the previous epidemics (SARS-CoV and MERS-CoV). The high variability region of the nsp3 protein was confirmed to exhibit the most variations between closest strains. It was then studied in the context of physiological and molecular data available in the literature. A number of convergent findings suggest de-mono-ADP-ribosylation (de-MARylation) of STAT1 by the SARS-CoV-2 nsp3 as a putative cause of the cytokine storm observed in the most severe cases of COVID-19. This may suggest new therapeutic approaches and help in designing assays to predict the virulence of naturally circulating SARS-like animal coronaviruses.

## 1. Introduction

Probably started in December 2019 from a zoonotic transmission from traded wild animals in a Wuhan market (Hubei Province, China) [1,2], the coronavirus disease 2019 (COVID-19) quickly reached a planetary dimension forcing billions of people into lockdown, grinding to a halt most of the world economies, and killing in excess of 375,000 of the 6.1 million people known to be infected (as of 2 June 2020). Initially catalogued as a respiratory disease (severe acute respiratory syndrome, SARS), the investigation of severe COVID-19 cases progressively revealed their association with a dysfunctional pro-inflammatory response leading to severe lung damages [3], but also cardiovascular complications [4] and multi-organ failures [5]. The unexpected severity and dimension of the COVID-19 pandemic prompted national research organizations to ask their scientists, even without prior knowledge on coronaviruses, to use their expertise to contribute to the solution of this formidable challenge. This work is part of this approach, that of a novice researcher in the field, but competent in viral genomics. In this paper, I present a bioinformatic investigation of publicly available sequence data, in an attempt to identify which molecular processes might be responsible of the specific clinical picture of COVID-19. Several coincidental findings examined in the light of the rich pre-existing bibliographical information strongly suggest that virus-induced de-mono-ADP-ribosylation (de-MARylation) of STAT1 might be one of the key steps leading to the inflammatory cytokine storm seen in the most severe COVID-19 cases.

## 2. Materials and Methods

Due to the lockdown constraints imposed during the COVID-19 epidemic, all the bioinformatics analyses presented in this article were performed on publicly accessible bioinformatic platforms. Databases searches were performed at NCBI using the online BlastP on various subsets of NR [6], structural analyses were performed using the PSIPRED Workbench at University College London [7], and sequence alignments at the EMBL-European Bioinformatic Institute with the Clustal Omega and Water services [8].

## 3. Results

As a simple, straightforward attempt to delineate genomic changes that might correlate with the crossing of the xenographic barrier to human, and with the clinical features of COVID-19, I compared the whole predicted proteome of SARS-CoV-2 with its closest animal-infecting relatives available in the NCBI public database. In a preliminary step, a comparison of the proteome sequences of 250 human-infecting SARS-CoV-2 isolates did not reveal any region of significant variability, as they were quasi-identical. For instance, out of 250 nsp3 protein sequences (1945 residues long), 214 were identical and 36 exhibited single amino-acid changes (99.95% identity), 11 of which were conservative (data not shown). One sequence from the group of 214 identical sequences (Accession # YP_009725299, Wu et al., unpublished) was randomly chosen and used for subsequent comparative analyses.

Variable regions became only detectable when comparing the polyprotein 1ab sequence of SARS-CoV-2 with that of its closest relatives in animal hosts. As previously noticed, the most similar isolate is bat coronavirus RaTG13 with 96.7% similarity (Accession # QHR63299) [1], followed by two other bat isolates (# AVP78030, # AVP78041, 93.5% and 92.5% identical, respectively) [9], and several similar pangolin isolates (e.g., # QIQ54046, 85.4% identical) (Cao et al., unpublished).

**Figure 1:**
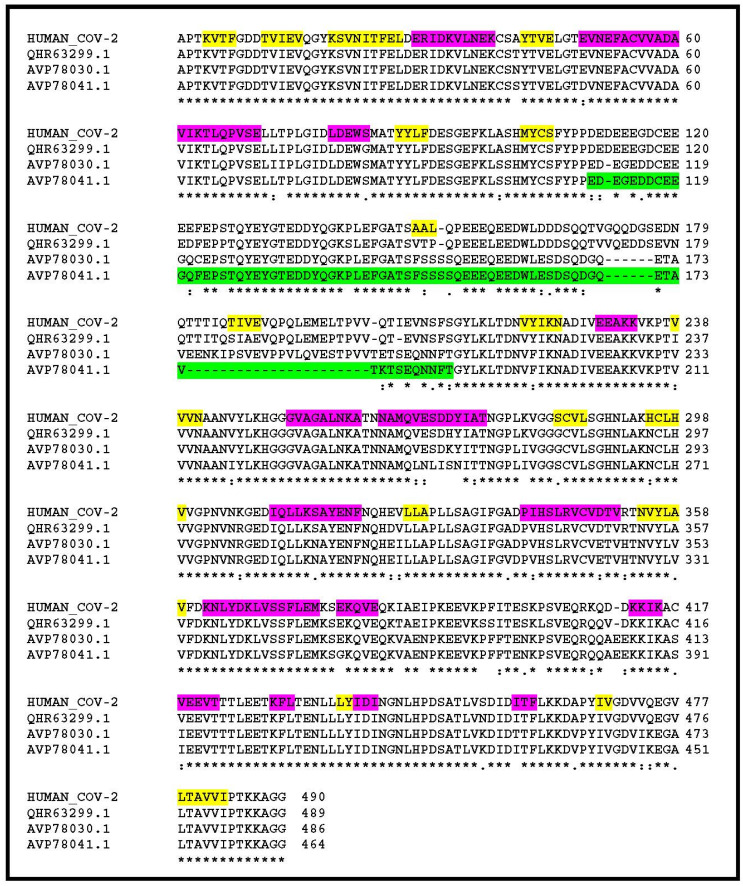
Multiple alignment of the human SARS-CoV-2 nsp3 N-terminal sequence and that of its 3 closest homologs in non-human-infecting coronaviruses. Sequences with accession #: QHR63299.1, AVP78030.1, AVP78041.1 are from 3 different isolates of bat-associated viruses. Their overall similarity over the entire SARS-CoV-2 nsp3 sequence are 93%, 83.3%, 80.9% identical residues, respectively. It goes down to 44% identity in the “variable region” highlighted in green. Predicted secondary structure elements are highlighted in yellow (extended) and purple (helix). The segment highlighted in green is predicted as intrinsically disordered. HUMAN_COV-2 corresponds to Genbank accession #: YP_009725299.

Looking for amino-acid changes occurring in clusters, my attention was drawn to a segment located near the N-terminus of the nsp3 protein (Figure 1). While 82.3% of its 1945 positions exhibit strictly identical residues between the human and bat homologs, this segment falls down to 44.5% identical residues. In SARS-CoV-2 nsp3, this segment is strongly enriched in acidic amino-acids and coincides with a paucity of predicted secondary structure elements (Figure 1). On the contrary, it is predicted as an intrinsically disordered protein region. Such domains form the basis of the dynamic “on–off” switch-type of interactions commonly found in signalling networks (reviewed in [10]). This stretch of amino-acids roughly maps to the hypervariable region (HVR) previously recognized upon the comparison of nsp3 homologs from more distant coronaviruses (reviewed in [11]), and to which no function has been assigned or predicted. I then focused further analyses on this well-delineated island of changes within the otherwise highly similar nsp3 from human, bat, and pangolin SARS-CoV-2 closest relatives.

**Figure 2:**
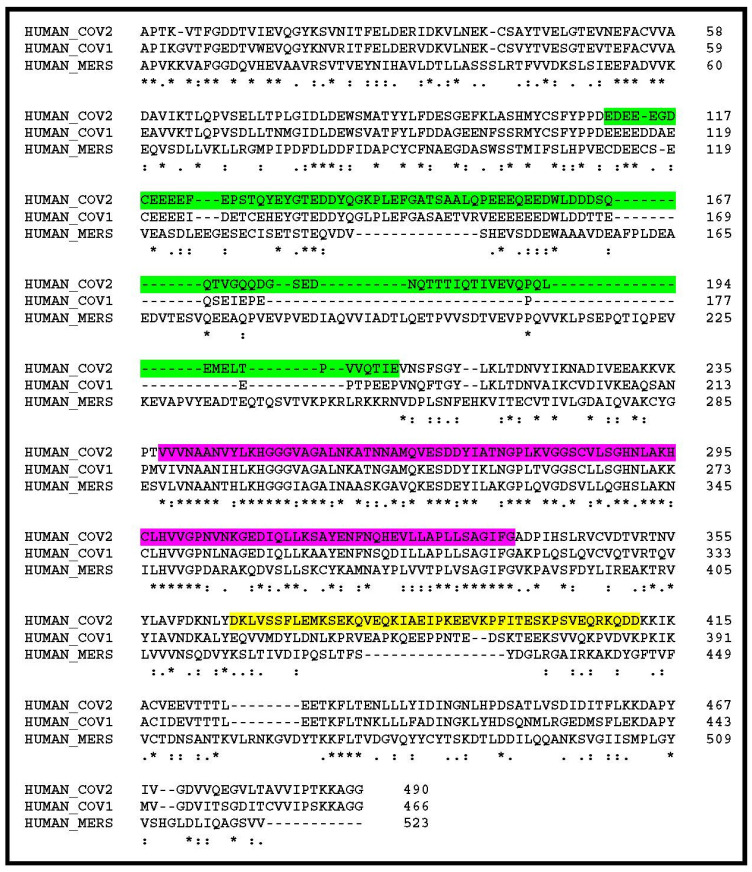
Comparison of the N-term regions of the SARS-CoV-2, SARS-CoV-1, and MERS-CoV nsp3 polyproteins. The glutamine-rich hypervariable region (HVR) is highlighted in green. The conserved ADP-ribose binding site is highlighted in purple. A region of lower similarity between SARS-CoV-2 and SARS-CoV-1 is also seen between positions 365 and 411 (highlighted in yellow). The rest of the SARS-CoV-2 and SARS-CoV nsp3 polyproteins are more than 80% identical, but less than 31% identical with that of MERS-CoV. Strictly conserved positions are indicated by “*”, conservative variations by “:” or “.”, insertions by “-”. Genbank accession numbers: CoV2: YP_009725299, CoV1: AAP13439, MERS: AGV08404.

The nsp3 segment, delineated as highly variable in the above comparison between the human SARS-CoV-2 and its closest animal homologs, is also the most divergent upon alignment of the human SARS-CoV-2 with the previous SARS-CoV-1 (responsible of the 2002 epidemics) [12], and even more dissimilar in the MERS-CoV (agent of the Middle East respiratory syndrome) [13] (Figure 2). In particular, multiple deletions and insertions make it unlikely that these homologous regions could adopt identical folds and perform similar functions.

The above analyses suggested that the N-terminal variable region (approximately from positions 111 to 237 in SARS-CoV-2) of the nsp3 protein, bearing the greatest variability between human-infecting strains, might be linked to the specific clinical pictures of the associated diseases.

Nsp3 is a large multifunctional protein, several domains of which have been mapped and characterized (reviewed in [11]). Starting from the N-terminus, there are in succession: an ubiquitin-like domain (positions: 20–106) known to bind to single stranded RNA, an ADP-ribose-binding module (positions: 238–337), a single-stranded poly(A) binding domain (positions: 678–743), a papain-like viral protease (positions: 746–1064), a nucleic acid-binding domain (positions: 1089–1201), and a seven-transmembrane G protein-coupled receptor C (positions: 1494–1563).

Interestingly, when the whole nsp3 sequence was compared to the entire human proteome using BlastP, the only reported significant matches (E-value < 10^−6^) were found to precisely encompass the ADP-ribose-binding module. Moreover, the top hits are human protein with a known function: all of them are mono-ADP-ribosyltransferases. This result strongly suggests that the ADP-ribose binding site predicted in the nsp3 protein is functional, as it has been experimentally verified in a number of other viruses, including SARS-CoV (reviewed in [14]). This is the first brick on which I will build my hypothesis: the N terminal part of the SARS-CoV-2 nsp3 protein is constituted of a variable protein-binding domain immediately adjacent to the highly conserved active site of an ADP-ribose (ADPR) processing enzyme.

**Figure 3:**
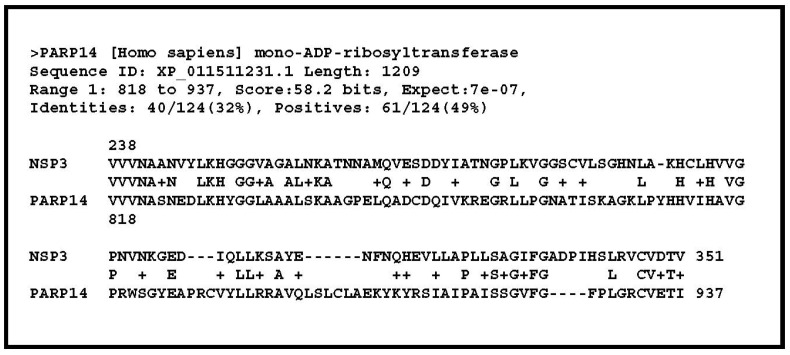
Best match from the BlastP similarity search output of the entire SARS-CoV-2 nsp3 protein (1935 residues) against the human proteome subset of the NR database. This unique match precisely corresponds to the ADP-ribose binding site, central to the viral macrodomain defined by previous structural studies [11].

Further inspections of the above BlastP output provided an exciting guess as to the putative target of the above ADPR enzyme. All the top matches of the nsp3 putative ADPR-binding site corresponded to that of various isoforms of the PARP14 protein (Figure 3). It turns out that this protein plays a significant role in the regulation of the inflammatory response that is out of control in severe cases of COVID-19.

PARP14 was shown to bind to a group of IFN-stimulated gene (ISG)-encoded proteins, most with an unknown function, and is required for their nuclear accumulation [15]. However, its best characterized function is to regulate macrophage activation via STAT1 mono-ADP-ribosylation (i.e., MARylation) [16].

STAT1 (for “signal transducer and activator of transcription 1”) is a member of the STAT protein family (i.e., STAT1-6), all members of which play some role in innate immune signalling. In response to cytokines and growth factors, these proteins are phosphorylated by the receptor-associated kinases, form homo- or heterodimers, and translocate to the cell nucleus. However, when it comes to signalling through any of the interferon (IFN) receptors, STAT1 is the most important [17].

The three different classes of IFN signal through distinct cell surface receptors. Binding of type I and II IFN to their respective receptors leads to the phosphorylation of STAT1 and STAT2 which then associate in heterodimers, recruit the IFN-regulatory factor 9 (IRF9) to form the IFN-stimulated gene factor 3 (ISGF3). ISGF3 then translocate to the nucleus were it to induce genes regulated by IFN-stimulated response element (ISRE). Alternatively, binding of type II IFN (IFN-γ) to its receptor complex lead to the sole phosphorylation of STAT1 that then associates in homodimers to form the IFN-γ activation factor (GAF) that translocate to the nucleus and activate genes regulated by the gamma-activated sequence (GAS) promoter elements. Through these distinct activation pathways STAT1 is involved in the regulation of different subsets of ISGs [18]. The STAT1 phosphorylation/de-phosphorylation process is thus central to the various immune responses triggered by distinct IFN sensing: antiviral defenses through the STAT1/STAT2 heterodimer formation (IFN I and III), or macrophage activation and proinflammatory responses through STAT1 homodimer formation (IFN-γ) (reviewed in [19]).

In the context of this complex interaction network, PARP14, hinders the phosphorylation of STAT1 at “Tyr 701” through the MARylation at “Glu-657” and “Glu-705”. This precludes the formation of the STAT1/STAT2 heterodimer or the STAT1 homodimers and their translocation to the nucleus. PARP14 activity is thus down-regulating the various IFN-triggered responses, in particular, the pro-inflammatory cytokine production in macrophages responding to IFN-γ stimulation [16].

This well-documented consequence of STAT1 MARylation on the inflammatory response, together with the presence of a PARP14-like ADP-ribose binding site flanking the variable region of nsp3, is at the basis of my hypothesis on the origin of the cytokine storm associated to the severe cases of COVID-19, detailed below.

## 4. Discussion

The ADPR-binding site that was identified in the SARS-CoV-2 nsp3 sequence corresponds to a conserved macrodomain (also called “X domain”) that follows the HVR in all coronaviruses [11]. The macrodomain is a ubiquitous structural domain that removes mono-ADP-ribose from proteins, reversing the activity of ADP-ribosyltransferases. This domain has been functionally characterized [14,20,21,22]. It was shown that the macrodomain from SARS-CoV (as well as that of other single-stranded positive-sense, single-stranded RNA viruses) can remove mono-ADP-ribose in vitro from a variety of targets, including PARP1, PARP10, and PARP15 [22], although no specific protein targets for viral macrodomains have been identified in vivo. Here I propose that STAT1 is the in vivo target of the SARS-CoV-2 macrodomain, thus precisely counteracting its mono-ADP-ribosylation by human PARP14.

The fact that the ADP-ribose binding site of the SARS-CoV-2 nsp3 macrodomain is most resembling that of PARP14 (Figure 3) suggests that they have a similar conformation, and are both compatible with the addition (for PAPR14) or removal (for nsp3) of an ADP-ribose moiety at the same site in the context of the STAT1 3-D structure.

However, the variable disordered region flanking the conserved macrodomain, a predicted protein-protein interaction domain, would exhibit a differential (or no) affinity for STAT1 in different SARS-CoV strains, hence modulating the efficiency of its de-MARylation by nsp3 and its consequence on the various IFN-triggered responses, including the proinflammatory activation of macrophages. Alternatively, the nsp3 variable region of SARS-CoV-1 or MERS-CoV could interact with different MARyled targets altogether, leading to different behavior vis-à-vis the host innate immune response [14,20,21].

According to this molecular scenario, the expression of nsp3 in IFN-γ-activated macrophages would indirectly promote a prolonged pro-inflammatory STAT1-dependent expression of interferon stimulated genes by inhibiting the downregulation of STAT1 through its MARylation and increasing the pool of STAT1 molecules available for phosphorylation. This would participate in the cytokine storm characteristic of severe COVID-19 cases.

Moreover, a recent unexpected finding makes the above hypothesis even more appealing. Following a monumental work, Ziegler et al. [23] just showed that the SARS-CoV-2 receptor ACE2 (Angiotensin-converting enzyme 2) is an interferon-stimulated gene in cell types found in the in the upper airway (epithelial secretory goblet cells), in the lung (type II pneumocytes), and in the small intestine (absorptive enterocytes), all locations and organs linked to clinical symptoms and viral shedding [4]. Accordingly, they found evidence for STAT1 binding sites in the promoter region of ACE2. In consequence, the higher phosphorylated/unphosphorylated ratio of STAT1 promoted by nsp3 in IFN-γ-stimulated macrophages might lead to more SARS-CoV-2 receptors being expressed at the surface of bystander epithelial cells, enhancing the infection process. Once started, a positive feedback loop will proceed, with more infections generating more IFN, activating more STAT1, in turn generating more inflammation and more virus receptors, with nsp3 permanently counteracting the PARP14-dependent MARylation of STAT1, thus precluding its downregulation and the termination of the pro-inflammatory phase.

Alternatively, MARylated (hence unphosphorylated) forms of STAT1 might have a protective role against viral pathogenesis that is counterbalanced by the SARS-CoV-2 nsp3 activity. Such a hypothesis is consistent with previous studies showing in a mouse model that SARS-CoV pathogenesis is regulated by STAT1 through an IFN-receptor-independent mechanism (reviewed in [24]). Unphosphorylated STAT1 (in the context of ISGF3) was shown to drive the constitutive expression of genes inhibiting viral replication [25]. In all cases, STAT1 remains the predicted target of SARS-CoV-2 nsp3 activity.

A relatively straightforward test of my hypothesis would involve the investigation of the ADP-ribosylation status of STAT1 during SARS-CoV-2 in vitro infection (using wild-type and macrodomain-mutated strains) and the experimental demonstration of a physical interaction between STAT1 and the relevant N-terminal regions of nsp3. As the decrease of the MARylated STAT1 pool could indirectly arise from the inhibition of the MARylation function of PARP14, the eventual interaction of the latter with nsp3 should also be investigated.

Finally, the discovery that ACE2 transcription is under the positive control of STAT1 [23] provides an elegant explanation for the conservation of a functional de-MARylation macrodomain, even though it was shown to be dispensable for the in vitro replication of the virus [21]. By following the natural incentive of all viruses to maximize their infectivity, hereby increasing the density of its receptor, SARS-CoV-2 triggers a cytokine storm as an unfortunate side-effect of COVID-19. Beyond the fundamental interest of establishing a link between nsp3 and the inflammatory response associated to the disease, an in vitro measurement of the de-MARylation efficiency of STAT1 by nsp3 synthetic constructs designed after new SARS-like coronavirus genomic sequences could help predict the virulence of wild animal strains before they start circulating within human populations. The identification of STAT1 as a target might also suggest some therapeutic avenues, such as the use of anti-IFN-y drugs (e.g., Emapalumab), or Jack1/Jack2 inhibitors (e.g., Baricitinib, Ruxolinitinib) (reviewed in [26]).
